# Pharmacological Profiles of Oligomerized μ-Opioid Receptors

**DOI:** 10.3390/cells2040689

**Published:** 2013-10-11

**Authors:** Cynthia Wei-Sheng Lee, Ing-Kang Ho

**Affiliations:** 1 Center for Drug Abuse and Addiction, China Medical University Hospital, Taichung 40447, Taiwan; E-Mail: T22529@mail.cmuh.org.tw; 2 China Medical University, Taichung 40442, Taiwan; 3 Graduate Institute of Clinical Medical Science, China Medical University, Taichung 40442, Taiwan; 4 Center for Neuropsychiatric Research, National Health Research Institutes, Zhunan, Miaoli County 35053, Taiwan

**Keywords:** μ-opioid receptor, bivalent ligand, receptor oligomerization

## Abstract

Opioids are widely prescribed pain relievers with multiple side effects and potential complications. They produce analgesia via G-protein-protein coupled receptors: μ-, δ-, κ-opioid and opioid receptor-like 1 receptors. Bivalent ligands targeted to the oligomerized opioid receptors might be the key to developing analgesics without undesired side effects and obtaining effective treatment for opioid addicts. In this review we will update the biological effects of μ-opioids on homo- or hetero-oligomerized μ-opioid receptor and discuss potential mechanisms through which bivalent ligands exert beneficial effects, including adenylate cyclase regulation and receptor-mediated signaling pathways.

## 1. Introduction

Opioids are one of the most commonly prescribed pain relievers and have been used to treat pain for thousands of years. Considered as broad-spectrum analgesics acting at multiple sites in both the central and peripheral nervous systems, opioids also have multiple side effects and potential complications [1]. Concerns regarding tolerance to analgesic effects result in a reluctance to prescribe opioids for pain management. Adverse gastrointestinal and central nervous system events, including constipation [2,3], nausea, vomiting and sedation, are responsible for a large portion of patients discontinuing opioid treatment, often leading to inadequate pain relief and poor quality of life [4]. Hyperalgesia (increased pain sensitivity), decreased libido, and other hormonal effects, and depression may also occur [5]. Controversially, opioids are uniquely addictive, leading to misuse and diversion [6,7,8,9].

Opioids produce analgesia via G-protein-coupled opioid receptors [10]. Three conventional opioid receptors—μ (MOR), δ (DOR) and κ (KOR) [11,12,13,14]—and a non-opioid branch of opioid receptors—opioid receptor-like orphan receptor (ORL1) [15]—have been characterized based on their pharmacological, anatomical and molecular properties, with ORL1 displaying pharmacology distinct from those of conventional opioid receptors [16,17,18,19]. Activation of MOR, DOR, KOR or ORL1 produces common cellular actions by regulating the same secondary messengers, including inhibition of adenylate cyclase (AC) activity [20,21,22,23,24,25,26] and N-type [27] and L-type Ca^2+^ channels [28]. Activation of opioid receptors also increases phospholipase C activity, causes a transient increase in intracellular Ca^2+^ [29,30] and activates inwardly rectifying K^+^ channels [31,32] and mitogen-activated protein kinases (MAPK) [33,34].

## 2. Oligomerization of μ-Opioid Receptor

### 2.1. The Roles of MOR in the Physiological Effects of Opioids

Opioids, such as morphine and methadone, mediate their physiological effects by preferentially activating MOR in neurons. The MOR agonists display the best antinociceptive activity, but also the highest abuse liability [35]. Disruption of the MOR gene leads to a complete loss of the main biological actions of morphine, including analgesia, reward, withdrawal, respiratory depression, immunosuppression and constipation, demonstrating that both therapeutic and adverse effects of the prototypic opioid results from its interaction with MOR gene products [35,36,37,38,39,40,41,42]. For example, morphine conditioning (repeated low-dose injections, 3 mg/kg, subcutaneously (s.c.)) that prolonged the time spent in the morphine-associated compartment in wild-type mice failed to induce place preference in mutant mice [39]. An analgesic dose of morphine (6 mg/kg, s.c.) increased respiration time and decreased respiratory frequency in wild-type mice, whereas no change in respiratory parameters were detected in similarly treated MOR-deficient mice [36]. A single dose of morphine (15 mg/kg, s.c.) inhibited gastrointestinal motility in wild-type mice, but no such change was observed in mutant mice at doses up to 35 mg/kg [41].

Desensitization and internalization of MOR are potential regulatory mechanisms contributing to the development of tolerance to opioids [43,44]. Activation of MOR by an agonist may result in receptor phosphorylation mediated by G-protein receptor kinases (GRKs). Subsequently, β-arrestins bind to the phosphorylated MOR, making this complex unable to couple to G-proteins to activate downstream effectors, resulting in receptor desensitization [45]. This MOR complex is recruited to the clathrin-coated pit and then removed from the plasma membrane by endocytosis [46]. Besides rapidly reducing the number of receptors present at the cell surface, endocytosis also mediates receptor “resensitization”, which involves delivering MOR to an endosome-associated phosphatase and then returning the dephosphorylated MOR to the plasma membrane via a rapid recycling pathway [47,48,49,50,51]. The degree of desensitization of MOR signaling observed in locus coeruleus (LC) neurons correlates well with that of agonist-induced MOR endocytosis assessed in HEK293 cells, further suggesting that desensitization and internalization are closely linked [52].

### 2.2. Pharmacological Responses of Oligomerized MOR

Opioid receptors are members of the rhodopsin family of G-protein-coupled receptors (GPCRs), which associate with each other to form dimers and/or oligomers [53,54,55]. Human MOR form sodium dodecyl sulfate (SDS)-resistant homodimers, and increasing concentrations and the longer exposure of agonists reduce the levels of MOR dimers with a corresponding increase in that of MOR monomers. This antagonist-reversible effect is suggested to be related to the endocytosis of MOR, since monomerization proceeds internalization [56]. MOR has also been reported to hetero-oligomerize with various receptors, including DOR [57], KOR [58], ORL1 [59], the somatostatin subtype 2A (sst_2A_) receptor [60], the substance P receptor (NK1) [61], cannabinoid CB_1_ receptor (CB_1_R) [62] and metabotropic glutamate receptor 5 (mGluR5) [63]. Receptor hetero-oligomerization usually leads to alterations in MOR phosphorylation, internalization, desensitization, MAPK activation and coupling to voltage-dependent Ca^2+^ channels [64]. Our group also demonstrated that methadone and buprenorphine, MOR full and partial agonists, respectively, exert initially different (acute), yet eventually convergent (chronic), adaptive changes of AC activity in cells co-expressing, presumably heterodimeric, MOR and ORL1 receptors [65]. Hetero-oligomerization of opioid receptors generates novel ligand binding properties, in some cases resembling pharmacologically-defined opioid receptor subtypes, but not unique opioid receptor genes [57,66,67]. How oligomerized MOR affects the effects of MOR ligands is the focus of preclinical research and might indicate a way for developing analgesics without unwanted side effects. 

#### 2.2.1. MOR-DOR

Early studies have suggested that MOR and DOR cross-talk and affect each other’s properties [68]. Morphine binds to MOR and DOR and inhibits neurotransmitter release [69]. In transgenic animals lacking MOR, DOR ligand-mediated analgesia is changed [70]. In DOR knockout mice, supraspinal DOR-like analgesia is retained and morphine tolerance is lost [71]. DOR antagonists-treated mice exhibit reduced development of morphine tolerance and dependence [72]. Reduction in DOR by antisense oligonucleotides attenuates the development of morphine dependence [73]. Ligand binding assays show that MOR-selective ligands inhibit the binding of DOR-selective ligands, both competitively and noncompetitively [74,75]. Radioligand binding and electrophysiological studies suggested that MOR and DOR colocalize in the dorsal root ganglia [76,77,78]. Immunohistochemical studies revealed that MOR and DOR colocalize at the same axonal terminals of the superficial dorsal horn [79] and ultrastructurally in the plasmalemma of the dorsal horn neurons [80]. Additionally, several neuroblastomas co-express MOR and DOR [81,82,83,84]. These lines of evidence suggest the physical existences of MOR and DOR complexes.

Heterodimerization of MOR and DOR have been demonstrated and may provide foundations for more effective therapies. Co-expression of MOR and DOR in heterologous cells followed by selective immunoprecipitation results in the isolation of MOR-DOR heterodimers [57]. The bioluminescence resonance energy transfer (BRET) assay showed that MOR and DOR conjugated in living cells [85]. A combination of MOR and DOR selective agonists synergistically binds and potentiates signaling by activating the MOR-DOR heterodimer [57]. Signaling by clinically relevant MOR ligands, such as morphine, fentanyl and methadone, can be enhanced by DOR ligands [85]. Furthermore, morphine-mediated intrathecal analgesia is potentiated by a DOR antagonist [85]. Morphine and [D-Ala^2^-MePhe^4^-Glyol^5^]enkephalin (DAMGO), traditionally classified as MOR selective agonists, selectively activate MOR-DOR heteromeric opioid receptors with greater efficacy than homomeric opioid receptors [86]. This is consistent with studies implicating the involvement of both MOR and DOR in analgesia, tolerance and dependence [71,72,73,87,88,89].

#### 2.2.2. MOR-KOR

MOR-KOR can form heterodimers with a similar affinity to that of MOR-DOR, as demonstrated by BRET, co-immunoprecipitation, receptor binding and G-protein coupling [58]. In males, spinal morphine antinociception requires the exclusive activation of spinal MOR; whereas in females, spinal morphine antinociception requires the concomitant activation of spinal MOR and KOR [90]. Expression of a MOR-KOR heterodimer is more prevalent in the spinal cord of proestrus *vs.* diestrus females and males. Spinal cord MOR-KOR heterodimers, likely to be the molecular transducer for the female-specific dynorphin/KOR component of spinal morphine antinociception, represent a unique pharmacological target for female-specific pain control [91].

#### 2.2.3. MOR-ORL1

MOR and ORL1 are coexpressed in the dorsal horn of the spinal cord, the hippocampal formation, the caudate/putamen [92,93,94] and several subpopulations of central nervous system (CNS) neurons involved in nociception [95]. Behaviorally, mice lacking the ORL1 gene partially lose tolerance liability to morphine analgesia [96] and show marked attenuation of morphine-induced physical dependence, manifested as naloxone-precipitated withdrawal symptoms after repeated morphine treatments [97]. Co-administration of ORL1 antagonist, J-113397, during conditioning facilitates morphine-induced conditioned place preference (CPP), and ORL1 knockout rats are more sensitive to the rewarding effect of morphine than wild-type control rats, suggesting that the ORL1 system is involved in attenuating the rewarding effect of μ-opioids and offers a therapeutic target for the treatment of drug abuse and addiction [98]. Functional interactions between MOR ligands and nociceptin, the endogenous ligand of ORL1, have been observed in the human neuroblastoma cell line BE(2)-C, which contains both MOR and ORL1 [99]. Immunoprecipitation assay demonstrated that MOR can physically associate with ORL1, resulting in a complex with a unique binding selectivity profile [100]. Furthermore, heterodimerization of MOR and ORL1 impairs the potency of MOR agonist, DAMGO, [59] and attenuates ORL1-mediated inhibition of N-type channels [101].

#### 2.2.4. MOR- sst_2A_

MOR and sst_2A_ receptors coexist and cooperate functionally in nociception pathways [102,103], and there is extensive cross-talk between opioid and somatostatin-mediated analgesia responses [104,105]. A particularly high degree of MOR-sst_2A_ receptor colocalization was observed in the locus coeruleus [60], a brain region involved in opioid withdrawal syndrome [106], and the attenuation of opioid withdrawal by the sst_2A_ receptor agonist octreotide may be due to the physical contacts of MOR and sst_2A_ in the locus coeruleus [107]. Co-immunoprecipitation studies provided direct evidence for the heterodimerization of MOR and sst_2A_. MOR-sst_2A_ heterodimerization did not substantially alter the ligand binding properties of the receptors, but selectively cross-modulates receptor phosphorylation, internalization and desensitization [60].

#### 2.2.5. MOR-NK1

MOR and NK1, the principle receptor for substance P, coexist and functionally collaborate in brain regions governing pain perception [108,109,110,111,112,113,114,115]. Moreover, MOR and NK1 are highly expressed in the nucleus accumbens, which mediates the motivational properties of drugs of abuse, including opioids. Interestingly, the rewarding effects of morphine are absent in NK1-deficient mice [113,114]. Using co-immunoprecipitation and BRET, MOR and NK1 were shown to form heterodimers [61]. MOR-NK1 heterodimerization does not significantly change the ligand binding and signaling properties of MOR, but dramatically altered its trafficking and resensitization profiles [61]. Altered β-arrestin trafficking in cells coexpressing MOR and NK1 could thus impact on opioid resensitization and the long-term cellular effects of opioids [61].

#### 2.2.6. MOR-CB_1_R

Opioids and cannabinoids share several pharmacological effects, such as antinociception, hypothermia, hypotension and sedation [116,117]. The synergy of the pharmacological effects of opioids and cannabinoids is attributed to the cross-talk between MOR and CB_1_R [118,119]. Furthermore, a CB_1_R agonist, delta 9-tetrahydrocannabinol, can enhance the potency of morphine [120]. MOR and CB_1_R colocalize in dendritic spines in the caudate putamen and dorsal horn of the spinal cord [121,122,123]. Coexpression of MOR and CB_1_R leads to MOR-CB_1_R heterodimerization, as revealed by BRET [124] and fluorescence resonance energy transfer (FRET) [62] assays. Guanosine 5'-O-(3-thiotriphosphate) (GTPγS) binding and MAPK phosphorylation assays demonstrated that MOR signaling is attenuated by CB_1_R agonist, and this effect is reciprocal in both heterologous cells and endogenous tissues coexpressing MOR and CB_1_R [124]. An electrophysiological analysis has also been developed to determine the functional coupling of the heterodimerized MOR-CB_1_R [62].

#### 2.2.7. MOR-mGluR5

MOR activity can be affected by presynaptic modulation through glutamate receptors. Glutamate elicits and modulates responses in the CNS via two groups of receptors, ionotropic (ligand-gated ion channels) and G-protein-coupled metabotropic receptors (mGluRs) [125,126,127]. mGluR5 plays major roles in modulatory CNS pathways and has pharmacological implications in pain [128,129]. Moreover, the inhibition of mGluR5 can modulate opioid analgesia and opioid tolerance. The specific mGluR5 antagonist, 2-methyl-6-(phenylethynyl)pyridine (MPEP), can block hyperalgesia and nociceptive behavior, and the co-administration of MPEP with morphine could suppress the loss of morphine-induced anti-nociception and inhibit the development of morphine-induced tolerance [130,131]. In human embryonic kidney (HEK) 293 cells coexpressing MOR and mGluR5, DAMGO-induced MOR phosphorylation, internalization and desensitization are attenuated by MPEP treatment [63]. Co-immunoprecipitation data further indicate MOR-mGluR5 heterodimerization [63]. Interestingly, the allosteric mGluR5 inhibitor MPEP is not simply acting as a blocker of glutamate signaling, but changes the conformation of MOR-mGluR5 heterodimer to affect MOR phosphorylation and desensitization [63].

## 3. Bivalent Ligands of Oligomerized μ-Opioid Receptor

Bivalent ligands are compounds that contain two recognition sites, or pharmacophores, joined through a connecting spacer. Pharmacophores are ensembles of steric and electronic features necessary for optimal supramolecular interactions with a specific biological target to exert its biological response. When talking about ligand-receptor interactions, pharmacophores are the molecular moieties of the ligands required for recognition by their corresponding receptors. 

The endogenous opioid peptide family is characterized by a common tetrapeptide sequence (Tyr-Gly-Gly-Phe) and comprises over a dozen ligands [132,133,134], including β-endorphin [135,136], enkephalins [137] and dynorphins [138]. Two additional endogenous ligands, endomorphin-1 and -2, with a tetrapeptide sequence (Tyr-Pro-X-Phe-NH_2_ X = Trp or Phe) different from that of classical opioid peptides have also been reported [137]. Increasing the distance between enkephalin pharmacophores results in DOR-selective compounds, while reducing the distance makes it more MOR-selective [139,140]. Synthesis of bivalent ligands has been one of the most promising ways of developing new opioid analogues since the 1980s [139,140,141,142,143,144,145,146].

Ligand-induced clustering of opioid receptors was observed on the surface of neuroblastoma cells [147]. This observation prompted scientists to design double pharmacophore ligands—bivalent ligands—as probes for bridging hypothetical dimeric opioid receptors [148]. Convincing evidence for homo- and hetero-dimers among opioid receptors has been presented since the late 1990s [149]. Portoghese’s group envisioned that a bivalent ligand with a spacer of optimal length would exhibit greater potency than that derived from the sum of its two monovalent pharmacophores [144,150]. Based on molecular modeling of interlocking transmembrane (TM) helices in a homodimeric MOR-MOR, it was proposed a decade ago that interlocking dimers with a TM5,6-interface between the 7TM domains may be the dominant form of dimers [151].

The recognition sites of opioid agonists and antagonists on dimeric receptors are believed to be separate. The MOR protection experiment was performed using MOR-selective agonists (morphine) and antagonists (naloxone) to determine their effectiveness in blocking irreversible MOR antagonism by β-funaltrexamine (β-FNA) in the guinea pig ileum preparation. Relatively high concentrations (0.5–1 μM) of MOR agonists were needed to protect MOR against inactivation, while antagonists in the low nM range were effective in blocking the irreversible effect of β-FNA. It was suggested that MOR activation and antagonism are mediated through separate negative allosterically coupled recognition sites in a dimer [152]. According to their theory, an opioid could modulate its own effects on opioid receptors via regulating its own concentration. At low concentration, single occupancy of the dimer would occur; at higher concentration, occupation of the second site would dampen the overall binding and activation of the opioid receptor through negative cooperativity. The frequently-seen bell-shaped concentration-response curve is consistent with this proposal, and exogenous antagonists were envisioned to have greater affinity for this second site [148]. Site-directed mutagenesis, combined with the classical structure-activity relationship approach, led to the identification of amino acid residues on opioid receptors and chemical groups on ligands participating in molecular recognition [148,153].

Several lines of evidence support the homo-oligomerization of MORs. The human MORs form SDS-resistant homodimers, and increasing concentrations and longer exposure of both peptide (DAMGO) and alkaloid (ohmefentanyl, etorphine and morphine) agonists reduce the levels of dimers with a corresponding increase in those of monomers [56]. Complementation of function after coexpression of pairs of nonfunctional MORs that contain distinct inactivating G-protein mutations linked to the C-terminal tails suggests that MORs exist as dimers [154]. This receptor homodimerization is facilitated by a cholesterol-palmitoyl interaction in the MOR complex [155]. The synthesis of homo-bivalent ligands of mixed KOR/MOR agonists/antagonists has been achieved [156,157,158,159,160,161]. However, none of the homo-bivalent ligands have been applied to study the homo-oligomerization of MORs.

Heterodimeric opioid receptors raised the issue of pharmacological selectivity. The transition from MOR to DOR agonism upon chronic exposure of mice to MOR agonists (methadone and heroin) might reflect changes in the distribution of MOR-DOR heterodimers [162]. If there is an increase of the MOR-DOR density, MOR agonists that bind to MOR-selective sites of the dimmers could be antagonized by the interaction of a DOR antagonist at the DOR-selective site in the heterodimer. The presence of heterodimeric receptors has important influence on the interpretation of experimental data and in the screening strategy using homogeneous populations of cloned receptors. Additionally, dimeric receptors may activate transduction pathways different from those elicited by monomers [148].

Bivalent ligands are not necessary bi-functional ligands, and *vice versa*. Bivalent ligands are compounds that have two pharmacophores with a long linker. Earlier studies on GPCR bivalent ligands did not aim to target dimeric complexes, and many such ligands have relatively short linking groups between the pharmacophores. This suggests that these bivalent compounds may be interacting with neighboring binding sites on a single receptor rather than bridging sites across a receptor dimer. In contrast, bi-functional ligands serve as agonists and/or antagonists for different monomer receptors because of their multiple functional groups within the molecule. Considering their activities of recognizing heterodimeric receptors and the pharmacological potential of being a new-generation analgesic, we also provided an example in this review.

### 3.1. MOR-DOR Bivalent Ligands

The cross-talk between MOR and DOR and its possible significance in morphine tolerance and physical dependence have been suggested [71,73,88,163,164,165,166,167]. The chronic effects of morphine can be blocked by DOR antagonists without significantly compromising its antinociceptive action [72,167,168,169]. Following the aforementioned findings, mixed MOR agonist/DOR antagonist ligands have been designed to develop analgesics devoid of the side effects of traditional opioids [170,171,172]. Bivalent ligands of opioid alkaloids [143,144,145,150,173,174,175,176] and peptide agonists [139,140,177,178] derived from enkephalins have been shown to have increased opioid receptor selectively and potency compared to corresponding monovalent counterparts.

Considering the evidence for MOR-DOR heterodimers in cultured cells [57,67,85], similar interactions may also occur *in vivo* to mediate the synergy of MOR and DOR agonists. Bivalent ligands containing MOR agonist and DOR antagonist pharmacophores, μ-δ agonist-antagonist (MDAN) series, have been designed to address the MOR-DOR interaction through which MOR agonist-induced tolerance and dependence are attenuated [87]. The pharmacophores were derived from the MOR agonist, oxymorphone, and DOR antagonist, NTI. A transition in the behavioral pharmacology is believed to be a function of the spacer length of the bivalent ligand that reflects the bridging of the MOR-DOR heterodimer. According to this theory, if the MOR-DOR heterodimers mediate tolerance and dependence at the molecular level, changes in tolerance and dependence as a function of spacer length could be a manifestation of bridging. Indeed, the bivalent ligand with the shortest length, MDAN-16, developed a certain degree of dependence, whereas the remaining members of the series with longer spacers were essentially without dependence development. When the spacer length was longer than 22 Å (MDAN-19 to -21), neither tolerance nor dependence was observed. It appears that bridging MOR-DOR heterodimers by MDAN ligands negatively modulates putative signal transducers, thereby reducing tolerance and dependence [87]. In HEK293 cells, MDAN-21 prevents endocytosis, while MDAN-16 gives rise to robust internalization, of MOR-DOR heterodimers [179]. This dramatically divergent internalization of MOR-DOR heterodimer elicited by MDAN bivalent ligands of different spacer lengths is relevant to the role of receptor internalization in tolerance [179].

### 3.2. MOR-KOR Bivalent Ligands

The bivalent approach was applied to synthesize mixed MOR-KOR ligands as potential analgesics with decreased side effect [180]. Compared to MOR agonists, KOR agonists lack respiratory depressant, constipating and strong addictive (euphoria and physical dependence) properties. However, clinical trials with KOR agonists have been aborted, because of the occurrence of unacceptable sedative and dysphoric side effects. It is now recognized that KOR agonists with some MOR activity produce fewer adverse side effects than highly selective KOR agonists, so the mixed MOR-KOR ligands might act as clinically useful analgesics [181].

A series of bivalent ligands containing a KOR antagonist pharmacophore, 5’-guanidinonaltrindole (5’-GNTI), and a MOR antagonist pharmacophore, β-naltrexamine (β-NTX), linked through a spacer of varying length have been synthesized and characterized *in vitro* [182]. The design rationale is based on the desire to maintain a favorable hydrophilic and lipophilic balance coupled with flexibility. This includes a spacer that contains (1) glycine units that maintain a favorable hydrophilic-lipophilic balance, (2) a succinyl unit that contributes to the flexibility for favorable interaction with heterodimers and (3) an alkylamine moiety attached to 5’-GNTI that permits variation of the spacer length by one atom increments. The antagonist activities of the bivalent ligands were evaluated by measuring the inhibition of Ca^2+^ release in HEK293 cells stably expressing KOR and MOR singly or simultaneously. The selective agonists used to evaluate the antagonism selectivity of the target compounds were U69593 (KOR) [183] and DAMGO (MOR) [184]. One bivalent ligand, KMN-21 (with a spacer length of 21 atoms), significantly antagonized both U69593- and DAMGO-induced Ca^2+^ release in cells containing coexpressed MOR and KOR [182]. It is noteworthy that the KOR-DOR bivalent ligand antagonist, KDN-21, also contains a 21-atom spacer, suggesting common bridging modes for MOR-KOR and DOR-KOR heterodimeric receptors [185].

Another monovalent bi-functional ligand, *N*-naphthoyl-β-naltrexamine (NNTA), which selectively activates heterodimeric MOR-KOR in HEK293 cells and induces potent antinociception in mice, has been synthesized [186]. In the mouse tail-flick assay, intrathecal (i.t.) NNTA produced antinociception that was ~100-fold greater that intracerebroventricular (i.c.v.) administration. No tolerance was induced by i.t. administration, but marginal (three-fold) tolerance was observed by i.c.v. administration. Neither significant physical dependence nor place preference was produced in the ED_50_ dose range. These results suggest an approach to potent analgesics with fewer deleterious side effects [186].

### 3.3. MOR-ORL1 Bivalent Ligands

Bifunctional MOR-ORL1 agonists are hypothesized to be useful as non-addictive analgesics or medication for drug addicts [187]. The concept of using mixed-action opioids for pain management and drug abuse treatment is clinically validated [188,189,190]. Buprenorphine, a MOR partial agonist and KOR antagonist, also has low efficacy at ORL1 [191,192]. Its ORL1 agonist activity is responsible for the attenuation of its antinociceptive activity at high doses [193] and the reduction of cocaine use in dually addicted cocaine-opioid addicts [194]. If ORL1 agonist-MOR agonist activities are present in the same molecule, the ORL1 activity may modulate the rewarding effects of the MOR activity, thereby producing opioid analgesics that have a reduced addiction liability. MOR-ORL1 agonists may also be used as drug abuse medications with diminished dependence and withdrawal tendencies.

SR16435 [1-(1-(bicyclo[3.3.1]nonan-9-yl)piperidin-4-yl)indolin-2-one], a nonselective ORL1 and MOR partial agonist, has potent antinociceptive activity in acute thermal pain, as well as CPP, an effect mediated by its MOR activity [195]. It is speculated that the partial agonist efficacy of SR16435 at ORL1 is not sufficient to attenuate the rewarding effect of its MOR activity. The *in vivo* pharmacological profile of three other ORL1 agonists, SR14150, SR16507 and SR16835, with different selectivity and efficacy for ORL1 and MOR, have been determined in a model of acute nociception (the tail-flick assay) and a model of reward (place conditioning paradigm) in mice [187]. SR14150 is a high-affinity ORL1 partial agonist that has 20-fold selectivity over MOR. It has antinociceptive activity by acting as a MOR partial agonist, but is not rewarding in the place-conditioning paradigm, due to its ORL1 agonist activity that attenuates MOR-mediated reward. SR16507 has high binding affinity for both ORL1 and MOR, acting as a full ORL1 agonist and partial MOR agonist. It has potent antinociceptive activity, but produces CPP. SR16835 is a modestly (seven-fold) selective ORL1 full agonist, with very low efficacy at MOR. It does not produce MOR-mediated antinociception and CPP. The overall antinociceptive and antirewarding profiles of these ligands depend on their selectivity between MOR and ORL1, as well as intrinsic activity at these receptors. Notably, the ORL1-selective ORL1-MOR partial agonist, SR14150, has antinociceptive activity without rewarding effects and may lead to clinically useful treatments for pain and drug addiction [187].

The structure-activity relationship (SAR) for discovering bifunctional MOR-ORL1 ligands, starting from ORL1-selective scaffolds, has been explored [196]. Based on the 2-indolinone class of ORL1 ligands [197], a 2-D pharmacophore model (comprised of three pharmacophoric features common to most ORL1 ligands) was developed to determine SAR leading to (1) selectivity *versus* the classical opioid receptors and (2) functional efficacy, *i.e.*, agonist or antagonist activity [198]. Most ORL1 ligands contain the piperidine ring, so the piperidine 4-position heterocyclic ring and the piperidine N-substituent were systematically analyzed. The compounds were tested for binding affinity at human ORL1, MOR, DOR and KOR transfected into Chinese hamster ovary (CHO) cells. Binding to the opioid receptors utilized the selective agonists, [^3^H]nociceptin, [^3^H]DAMGO, [^3^H]Cl-DPDPE (d-penicillamine(2,5)-enkephalin) and [^3^H]U69593, for ORL1, MOR, DOR and KOR, respectively. Functional activity was determined by stimulation of [^35^S]GTPγS binding to cell membranes [192,199,200]. Data from these 17 compounds indicate that modulation of the ORL1 agonist *versus* MOR agonist potency using SAR approaches will be useful for developing bivalent ORL1-MOR ligands for medication development [196].

### 3.4. MOR-mGluR5 Bivalent Ligands

The selective mGluR5 antagonist, MPEP, acts allosterically by binding to the 7TM domain of the receptor [201]. Co-administration of MPEP and morphine could enhance morphine antinociception and suppress morphine-induced tolerance and dependence, suggesting a design strategy for developing potent analgesics targeting both MOR and mGluR5 [130,202,203]. The bivalent ligands being synthesized [204] contain pharmacophores derived from the MOR agonist, oxymorphone, and the mGLuR5 antagonist, *m*-methoxy-MPEP (M-MPEP) [201], linked through spacers of varying lengths (10–24 atoms). The series of compounds was evaluated for antinociception using the tail flick and von Frey assays in mice pretreated with lipopolysaccharide (LPS). MMG22 (22-atom spacer) is the most potent member of the series (intrathecal ED_50_ (effective dose in 50% of the population) ~9 fmol). Since other members with shorter or longer spacers have ≥500-fold higher ED_50_s, the exceptional potency of MMG22 may result from the optimal bridging of the MOR-mGluR5 heteromer. MMG22 possesses a >10^6^ therapeutic ratio, suggesting that it might be an excellent candidate for the management of chronic, intractable inflammatory pain via spinal administration [204].

## 4. Conclusions

Bivalent ligands of the opioid receptors have been prepared to improve the pharmacological properties of opioid ligands, because ligand-induced clustering of opioid receptors occurs on the cell surface [142,147,148,205]. These efforts have been validated with the characterization of homo- and hetero-oligomers of the opioid receptors [55,57,67,85,206,207]. Hetero-oligomers of MOR with other receptors display altered ligand-binding profiles and novel signaling properties relative to receptor monomers [55,206]. Bivalent opioid ligands capable of interacting simultaneously with different recognition sites in the hetero-oligomerized MOR complexes exhibit increased efficacy in signal amplification [205,206], thereby with (1) enhanced agonist or antagonist activity, (2) improved metabolic stability, (3) improved membrane permeability, (4) reduced opioid-induced tolerance, (5) diminished physical dependence and (6) a raised potential for the treatment of drug addiction [205].

The oligomerization of GPCRs is dynamic and regulated. Microscopically, bivalent ligands bind to and thereby stabilize or de-stabilize pre-existing dimers. Specific ligands may promote ligand-induced dimerization or inhibit further dimerization, depending on the intrinsic properties of the ligands. The pharmacological difference between bivalent ligand binding and co-application of two monovalent ligands is that the former interact with two adjacent receptors simultaneously, but the latter bind to two distant receptors sequentially. The downstream signaling process of these two events could be different spatially and temporally. The advantages of using bivalent ligands as therapeutic tools are less undesired side effects, whereas the disadvantages are non-specific and unexpected effects where the targeted heterodimers do not colocalize. Whether both ligand binding sites are equal in a dimer or have a certain degree of cooperativity depends on the structures of heterodimers and the orientation of the linked pharmacophores on the bivalent ligands. We speculate that most of the binding sites are not equal in the heterodimers and are somewhat cooperative when interacting with the bivalent ligands.

There are caveats that one should be aware of when evaluating the data of the oligomerized opioid receptors. Most studies for oligomerization employ *in vitro* methods of overexpression receptors in cell lines. The overexpression system apparently will have artificial results by forcing receptors together. Co-immunoprecipitation methods contain detergent, which could disrupt protein-protein interactions, thus disrupting receptor oligomerization. Moreover, the *in vivo* data are not solid for receptor oligomerization, and most of the studies are based on a heterologous expression system. The observed pharmacological profiles of the bivalent ligands linking a relatively non-selective agonist and an antagonist *in vivo* might not always reflect the effects of the heterodimers. In areas where the distributions of the two receptors are non-overlapping, the effects of the ligands observed may be owing to the agonistic or antagonistic effects on one of the receptors alone. For example, Pickel and her co-workers reported that mu-receptors were located in 21% of the dendritic profiles and 3% of the axon terminals containing CB1 receptors in the rat nucleus accumbens shell [123], a rather low percentage of neurons that express both receptors in question. Although their findings provide ultrastructural evidence that cannabinoid-opioid interactions may be mediated by activation of CB1 and MOR within the same neurons in the nucleus accumbens, for ligands that are not selective for heterodimers, their pharmacological effects actually reflect the activities of heterodimers and the homodimers or monomers in combination *in vivo*.

The high-resolution crystal structures of MOR, DOR, KOR and ORL1 in ligand-bound conformations have been resolved [208,209,210,211]. The MOR structure shows tightly coupled pairs of receptor molecules, held together predominantly by complementary interactions involving TM5 and TM6. This pairing might regulate MOR signaling [211,212]. In contrast, the KOR structure shows a dimeric arrangement involving interactions of TM1, TM2 and helix 8 (H8), similar to the alternative, less compact packing in the MOR structure [209,212]. The proposed roles of the TM5-TM6 and TM1-TM2-H8 interfaces in functionally relevant receptor-receptor interactions need to be addressed to reveal the role of oligomerization in the signaling of opioid receptors [212]. The mission of identifying the functionally relevant oligomerization interfaces continues and will provide further insight into the SAR of the bivalent ligands and the oligomerized opioid receptors.
